# Hospital and Institutional News

**Published:** 1915-06-26

**Authors:** 


### HOSPITAL AND INSTITUTIONAL NEWS.

### THE NEW SECRETARY OF HAMPSTEAD GENERAL HOSPITAL.

THE successful candidate for the post of secretary to the Hampstead General Hospital is Mr. J. Byrne, who was appointed as a result of the council meeting last week. He is understood to have had no previous hospital experience, and in this connection it will be recalled that, on the present occasion, hospital experience was not insisted on in the widespread advertisements which invited applications for the post. About two years ago Mr. Byrne was invalided out of the Indian Civil Service. As we were asked to publish a statement to the effect that the applications were so numerous that individual acknowledgment was necessarily delayed, it may be added that the number of applicants for forms was in round numbers 500, and the number of those who completed the forms 340. As we noted last week, the short list of ten was reduced to three, and the two candidates besides Mr. Byrne were Mr. Ian McKay, assistant secretary at the Chelsea Hospital for Women, and Mr. Madge, of the West London Hospital. The appointment shows that the council of the Hampstead General Hospital are pursuing the policy, which led to Mr. Kyle's appointment, of choosing a secretary without previous hospital experience.

### THE MEDICAL SERVICE OF THE GERMAN ARMY.

DETAILS of new developments in German military surgery are difficult to obtain, but occasionally minor matters are heard of through neutral sources. For instance, the Berlin correspondent of an American contemporary sometimes refers to the question. The new methods of warfare, as he points out in a recent letter, have brought new requirements for the equipment of the medical service. For example, there are now special first-aid packages for aviators, and special litters for trench work. These litters are made so narrow that they can easily be carried through the narrow trenches. They have handles on all sides, and can be strapped on the back, so that a single person can thus carry a wounded soldier on his litter. Knapsacks fitted with surgical supplies are also provided; the drugs used are in tablet form, or in sterilised solutions in small fused vials. According to the same correspondent, the central headquarters for the supplies for the entire sanitary service of the Army and Navy are installed in the Kaiser Wilhelm Military Medical Academy at Berlin. The new goods as received are stored in the broad entrance-hall. After they have been carefully inspected and approved they are arranged on rows of shelving, which are installed through the rooms on the various stories. On these ranges of shelving are arranged all the apparatus, instruments, dressing materials, and other things required for the medical and surgical work, everything being stored in such a way that it is instantly available. During the winter the trucks were loaded with supplies in a heated shed to avoid injury of articles that might have been damaged by the cold.

### THE ST. GEORGE'S HOSPITAL SITE.

THE meeting of the Court of Governors of St. George's Hospital, held last week, had before it a report of the house committee, in which the future of the hospital was outlined. The report recalled that the Hospital Bill to empower the sale of the hospital site and to provide for agreements with the governors of Westminster Hospital for amalgamation and removal of the two hospitals to a new site had received the Royal assent last July. The committee reported that although the site at Hyde Park Corner had been for sale for several years before the outbreak of war, yet no sum sufficient to warrant removal had been obtained. The report then proceeds: "It is now obvious that many years must elapse before there can even be a hope of obtaining an adequate price. This being so, the committee are about to consider the question of making provision for bringing the hospital up to date on the present site." According to a statement made by Mr. F. J. Frankau, who presided, the hospital is to remain in the neighbourhood, which he thought would redound to its ultimate benefit.

### SOLDIERS' DIET IN THE COVENTRY HOSPITAL.

A LETTER in the local newspaper appealing for vegetables on behalf of the Coventry and Warwickshire Hospital was made the subject of discussion at the monthly meeting last week. Mr. H. H. Ilett asked if this letter was the outcome of a conversation he had had with the Chairman, Mr. W. G. Wormwell, in which he remarked that the soldiers complained of a lack of green vegetables. The speaker added that it was most important that it should not be asserted that the diet for the wounded in Coventry Hospital compared unfavourably with that in the military hospital. He asked if the addition of green vegetables was to rest on the response to the Chairman's appeal. It seemed odd that the soldiers could get plenty of butchers' meat, which was so dear, and could not get green vegetables, which, he believed, were cheap. The Chairman replied that the dietary was always before the management. The present matron had introduced considerable reforms in the dietary from the patients' point of view, by which the whole hospital was affected. They had 160 patients, and the matter was not so simple as Mr. Ilett thought. According to a recent resolution, of which Mr. Ilett seemed unaware, the soldiers are having green vegetables three times a week. The Chairman added that in a sense they were experimenting. They would try vegetables three times a week, hence their appeal to the public to help them in the trial. The implication of this discussion is that vegetables even three times a week is something of an innovation. The just inference is that the present dietary scale at this hospital is seriously defective.

### LEICESTER WORKMEN AND THE WOUNDED.

A very pleasant ceremony took place at Leicester on Saturday last in one of the new wards of the military base hospital, when the Mayor (Alderman J. North), on behalf of the town, thanked the men who had been engaged on the extension of the building for the hearty way in which they had responded to the appeal made to them to complete the work at the earliest possible moment, and in the name of the contractors, Messrs. Henry Herbert and Sons, handed to them £150 earned in bonus by the builders for having completed the work fifteen days under the stipulated time. The contract was to build ten new military wards. The Government originally allowed twelve weeks for the completion of the contract, but Messrs. Herbert and Sons undertook with the architect, Mr. Perkins Pick, to their infinite credit, to finish the work in ten weeks. This undertaking was made subject to a bonus of £10 per day for every day under time, or a fine of £10 per day for every day over time. The firm then placed the matter before their employees, and promised that any money earned by doing the work under time should be handed over to them, plus overtime money. The Mayor, who had keenly interested himself in the matter, saw the officers of the trade societies concerned, and arranged that Messrs. Herbert should have plenty of labour, and that any overtime necessary should be worked. The men worked splendidly, and had the satisfaction of earning £150 in addition to their ordinary wage. The Mayor, in making the presentation, said he was particularly pleased, because it took some persuasion to induce the War Office to change their plan of commandeering the De Montfort Hall and some six or more of their principal elementary schools. The men who had assisted in the work had shown their patriotism as truly as men engaged in other work for the country, and he thanked them on behalf of the town.

### POSTPONING EXTENSIONS TILL AFTER THE WAR.

Notwithstanding the resolve of the Allies to bring the war to a successful conclusion at any sacrifice, there is a common assumption that the cessation of hostilities, with the success of our arms, will at once allow the energies which are now being deflected from their normal course to return to their ordinary channels. We know that in the sphere of diplomacy an immense amount of work will be thrust upon us the moment peace is declared; but we are less apt to foresee the inevitable delay that will equally occur before we can resume our normal activities. Thus we often hear of hospital extensions being postponed till after the war, and it is therefore necessary to remind ourselves that such a postponement may last longer than hostilities. Thus at a recent discussion at Dundee on the advisability of proceeding at once with the erection of two phthisis pavilions at King's Cross Hospital, a speaker pointed out that not only did the needs of the city actually demand the new accommodation now, but also that prices even after the war was over would remain as they are for a considerable time. Two other speakers endorsed this view, and one added that the Government embargo on spending money was never meant to preclude the undertaking of expenditure for necessities. As we believe that, under proper direction, the popularity of the hospitals through the war work which they have undertaken more than counterbalances the difficulty of raising money for charities, the moral seems to be that no committee need hesitate to appeal for money for hospital purposes, provided that the end in view is urgently essential.

### THE OUT-PATIENT PROBLEM AT WOLVERHAMPTON.

The shortage of doctors at Wolverhampton led, early in May, to the closing of the out-patient department at the General Hospital, so far as medical cases were concerned. At a meeting of the Hospital Saturday Committee, however, Mr. W. H. Harper, the hospital secretary, announced last week that, as a result of several meetings and conferences with the medical staff, it was recommended that the department be reopened. A stipulation is, however, that only those patients will be seen who have been recommended by a medical practitioner. A hospital note, as usual, will have to be produced. Mr. Harper explained that this was the best that the hospital could do, and that the present arrangement was made because the Board's wish that the department should be open on the old conditions had been found impracticable. The Saturday Committee welcomed the proposal, and expressed their gratitude for the work performed by the depleted medical staff, who, it will be realised, are now responsible for their own work, that of their colleagues absent on active service, and the hospital's also.

### NERVE CASES IN THE ARMY.

The Under-Secretary for War was asked last week the names of the clearing houses to which soldiers of the rank and file disabled by nerve strain were sent on arrival from the Front, and the names of the military hospitals specially adapted for the treatment of uncertifiable cases. In his reply Mr. Tennant pointed out that the clearing houses were the Royal Victoria Hospital, Netley, and the 4th London (Territorial Force) General Hospital, Denmark Hill. These cases were sent to various military hospitals throughout the country, and special neurological sections had been established in connection with the Territorial Force general hospitals, so as to bring the patients under the observation of the eminent neurologists at these centres. It was not possible, Mr. Tennant added, with any degree of accuracy to give the number of "border-line cases." The Red Cross Military Hospital, Maghull, and the Springfield War Hospital were taken over as military hospitals through the Board of Control, and the superintendents of these institutions were appointed to the charge of the military hospitals when they were taken over. For the time they were purely military medical officers. In answer to further questions he went on to say that the cases of nerve-shock to which the word "transient" might be applicable would be sent to military hospitals. Neither the desire nor the intention was lacking in his military medical advisers to save the soldiers and their families from the opprobrium associated with having been treated in a lunatic asylum.

### REGIMENTS AND THE COUNTY HOSPITALS.

Any sign of the normal extension of hospital activities is gratifying at the present time, and an example of this is the laying of the foundation-stone of the new out-patient department at the Royal Albert Edward Infirmary, Wigan. The new buildings, which are the local memorial to the late King, are to cost £12,000, of which rather more than half —£7,700—has been subscribed. As there were over 11,000 out-patients last year, each of whom made on the average four attendances, it is not surprising that the accommodation of the original institution, which was built in 1873, has long proved insufficient for its work. It has been suggested that the remainder of the money required should be raised as a special acknowledgment of the bravery and determination of the Lancashire Territorials, whose landing at a difficult place on the Gallipoli peninsula has led to the spot having been christened the "Lancashire Landing." There is an appositeness in this which may well appeal to the population of Wigan and the neighbouring districts which the hospital serves. So far as we know this is the first attempt to associate hospital schemes with the acts of heroism on the part of troops at the Front whose regiments are named after the county in which the hospital wishing to commemorate their deeds is situated.

### THE NEW WING AT HASLAR.

Thanks to the generosity of the women of Canada, a fine new wing is being built in the grounds of the Royal Naval Hospital, Haslar. Soon after the declaration of war the women of Canada raised a fund of £50,000 to aid the nursing of British sick and wounded, which was handed over to the Imperial Government to use as their information decided. As an extension at Haslar had long been wanted, most of the money was devoted to this purpose, and the patriotism of the givers will be acknowledged by christening the new buildings the Canadian Women's Wing. Naturally, the number of sick and wounded received since the outbreak of war has been much increased by the expedition against the Dardanelles, and consequently the new wing, which sets free 250 beds in the main building, will prove a most valuable addition to the hospital. It is, indeed, hardly correct to speak of the new wing as an addition, since its value lies in allowing certain beds, originally intended to form part of the patients' quarters, but actually occupied by the nursing staff, to revert to their proper use. The new wing will form a complete unit, with its own kitchen, recreation rooms, and gymnasium, for the staff. In fact, the staff will now have better quarters, and quarters more worthy of the standard prevailing throughout the rest of the building. The new wing is to consist of three floors, with a verandah along its whole length, and it is expected that the work will be completed before Christmas.

### HOSPITALS AND THE COAL SUPPLY.

The main sociological effect of the war has been the increased amount of State control of industry and business on lines which would have been most probably criticised in time of peace. That State action, however, is often demanded in new fields the following letter from the Treasurer of the Royal County Hospital, Winchester, will show. It appeared in the *Morning Post* last week: "Can the Government do nothing," Mr. Leonard Keyser writes, "in the way of taking the coal supply in hand? The increase in price is positively alarming. To give our experience at this hospital. Our present contract for steam coal is £1 6s. 6d.; this contract terminates at the end of the month. The same firm state that after July 1 they will be obliged to charge an increase of 13s.; that will mean to us 65s. a week extra expenditure. With all these increased prices pressing on them one after the other, the immediate future of voluntary hospitals must cause the deepest anxiety."

### THE EXCHANGE OF MEDICAL OFFICERS AND DISABLED PRISONERS.

It is satisfactory to record the reply given by Lord Robert Cecil in the House of Commons to a question concerning the exchange of British and German military medical officers. Asked whether an agreement had been reached regarding such an exchange, Lord Robert replied that this was so, and that it was hoped that the agreement would shortly be made effective. He explained that some delay had been caused owing to the necessity for the military authorities to determine exactly the members of the British and German military medical *personnel* who are entitled to be exchanged. As we pointed out some time ago, it is so much to the interests of both belligerents that military medical officers should be exchanged that it was inconceivable that an arrangement could not be come to on the point. On Tuesday last, moreover, Lord Robert Cecil stated that an exchange both of sanitary *personnel* and of incapacitated prisoners of war had just been finally arranged with the German Government through the United States Embassies at Berlin and in London, and would be carried out early next week.

### TRANSFERS TO THE ROYAL ARMY MEDICAL CORPS.

The much-discussed need for so reorganising our military and industrial resources that every man will be in a post for which he is most fitted is beginning to be acted upon. Thus it is understood that instructions have been issued by the War Office to the effect that medical men, who at the present time are serving with the combatant ranks, may obtain immediate transfer to the Royal Army Medical Corps. Application for transfer will pass through the hands of the commanding officers. This action is a step in the right direction, and may even prove sufficient; but it is not so drastic as the regulation understood to have been made and carried out in the French Army, by which the munition factories have already been replenished by every man in the Army who had previously obtained proficiency in ammunition-making. In this connection we may note that the enlistment of married men in this country does not seem to be exercising the War Office or the Government, since the replies which Mr. Tennant has lately given in the House of Commons would seem to indicate that they regard it as a feature of our splendid voluntary system with which no interference is desirable. Without entering on a delicate discussion of no immediate interest to hospital men, it may be remarked that the means of securing transfer from the combatant ranks to the R.A.M.C. do, however, indicate that the question of each man being in the best place for him is beginning to receive practical attention. Once begun, it would be rash to say at what point the process will stop.

### SOMETHING WORTH SEEING.

One of the most interesting and inspiriting works for cripples and others is the Heritage Colony at Chailey, Sussex. It is to have a field-day on July 1, when H.R.H. Princess Louise, Duchess of Argyll, will inspect the military wards named after her at the Heritage Colony. This inspection will give a welcome opportunity to all who can appreciate really good work which should not be missed.

### THE MENTAL HOSPITAL ROLL OF HONOUR.

Reference has already been made in these columns to the excellent way in which the staff of the London County mental hospitals have responded to their country's call. A report just issued by the London County Council includes the names of three of the mental hospitals' staff who have laid down their lives. They are Albert Seal, attendant at Banstead, private, 2nd Battalion, Scots Guards, who died after being invalided home from France; William West, first-class attendant at Hanwell, private in the Royal Marine Light Infantry, who was killed in action near the Dardanelles; and Benjamin Hambley, second-class attendant at Long Grove, private, 17th (Duke of Cambridge's Own) Lancers, attached 2nd Life Guards, who was killed in action. In this connection it is understood that the County Council have agreed that in selecting persons for employment in its service after the war due weight should be given to the claims of such candidates as have rendered, or attempted to render, national service.

### INDIAN RULER'S GIFT OF A HOSPITAL FOR OFFICERS.

A list of the private residences which have been given as hospitals for wounded officers would make an interesting postscript to a medical history of the war. A residence deserving of honourable mention in it would certainly be "Jamnagar," Staines, which the Jam Sahib of Nawanagar, K. S. Ranjitsinhji, has presented to the King for this purpose. Now to be named the Prince of Wales' Hospital, and to be supervised by the War Office, the new institution is to be maintained entirely by the donor in conjunction with the Maharaja of Kashmir and the Maharaja of Patiala. The thoroughness with which every detail has been remembered by his Highness is apparent from his gift of an ambulance and of two motor-cars which he has provided for the use of officers during their convalescence. The accommodation is divided into eight wards, and the total number of beds is forty. It does not require much insight to feel that the occasion of the opening, which took place on Friday last week in the presence of the Jam Sahib of Nawanagar, was of unusual interest. Its importance, indeed, is apparent in the remarkable speech that he made, which contained a passage to the effect that he had never been so proud of being an Indian as when the King-Emperor had allowed Indian troops to fight side by side with the English. The opening ceremony was performed by General Sir O'Moore Creagh, late Commander-in-Chief in India.

### WOMEN AND THE HEALTH OF THE CIVIL POPULATION.

Both directly and indirectly the conditions inseparable from war exercise an adverse influence on the health of the civil population. Not only is the natural resistance to disease impaired by anxiety, excitement, and altered home conditions, but the facilities for the transmission and development of diseases due to infective micro-organisms are increased, so that a higher death-rate from infectious diseases results. The health of the civil population, more especially that of infants and children in some parts of the country, gives cause for anxiety, as indications point to a dry, hot summer. At such a critical stage as the present special precautions are called for, and those responsible for adequately carrying out these precautions are the public health authorities and the people themselves. The chief danger of the summer of 1915 lurks in dust and flies. Money spent by local authorities in efficient scavenging and the frequent removal and destruction of house refuse should be regarded as a premium paid against loss from disease and death. In the home, cleanliness, fresh air, the protection of food from dust and flies, the removal or destruction of all house refuse and garbage, the sterilising of milk, and, where necessary, of water, and the whitewashing of outhouses, if efficiently carried out, will reduce to a minimum the risk of serious epidemic disease during the coming months. The truth is that in the present national crisis the women of England have their part to play no less than the men at the Front.

### A MODEL OF PAPER AND PRINTING.

We are always glad to draw attention to any annual report which presents attractive features, or original ideas, and when we do so it is generally to record some novelty, such, for instance, as the introduction of coloured illustrations, which we had occasion to mention a little while ago. It is a pleasure to us, therefore, to mention as highly worthy of praise the latest (116th) annual report of the Liverpool Royal Infirmary, which is one of the most attractive reports we have observed for a long time. Its most original feature, however, is that there is no original feature in it. It depends entirely for its delightful effect simply on the care and taste which have chosen the paper on which it is printed and the type in which it is set. The first thing one notices is the lightness in the hand of this quarto volume, in its simple grey cover, with red lettering above and below the hospital's seal. Turning the pages, the wide margins and neat type make the report look very inviting, while even the "form of bequest" and the list of subscribers become pleasant to look at from the artistic arrangement and pagination of the report. It is a revelation of how much printing and paper can achieve; and simplicity and good taste are apparent in every line of it. These features mean money, for they ensure that every page of such a report will be examined with interest. We heartily congratulate Mr. F. H. Moore, the superintendent, on a report of which any institution may well feel proud.

### A RECONSTITUTED SANATORIUM.

At the beginning of this month King Edward VII. Sanatorium, Midhurst, returned to its original function, which had been interrupted by the war. It will be remembered that early in the year a request from the War Office was acceded to, and fifty beds were set apart for the admission of soldiers who became affected with tuberculosis. The carrying this into effect necessitated the reservation of the female wing of the sanatorium, amounting to half its accommodation. The plan proceeded until the Insurance Commissioners decided to make their own arrangements for the reception of non-commissioned officers and men suffering from tuberculosis, most of whom are under the Insurance Act. This decision meant the removal of those in the sanatorium to institutions corresponding to the localities in which the patients intend to reside. King Edward VII. Sanatorium is, therefore, able to resume its work for those male and female patients, in professional and kindred employments, whose limited means put difficulties in the way of their receiving the necessarily extended institutional treatment which pulmonary tuberculosis requires.

### MEDICAL INSPECTION AND ANÆMIC SCHOOL CHILDREN.

Since the various branches of the medical service at home have been interfered with as a consequence of the war, it is important to keep in mind the activities which are still going on lest we should be tempted to acquiesce in their diminution, and get used, so to speak, to going without. Thus several interesting points are contained in the annual report relating to the medical inspection of school children at West Bromwich, which has been issued by the school medical supervisor, Dr. Woolsey Stocks, and the school medical officer, Dr. Jessie Valentine. They report that a marked increase has taken place in the extent to which defects have been followed up. They note, however, a very large number of cases of defective eyesight among children old enough to leave school. Girls appear to be worse sufferers than boys in this respect, and it is suggested that this may be due to the greater amount of close work done by them. The girls, again, show a degree of anæmia, which is attributed largely to poor feeding, and the writers regret that it is not within the scope of the medical service to treat them, since a timely tonic would prevent debility in many cases. The case for special schools is once again emphasised from experience with defective children; but in the case of the anæmic boys and girls we are forced to the old conclusion that the real stumbling-block is economic, in other words, poverty, to which underfeeding and the anæmia consequent on it are directly due. That is why the school medical service is of such value in that, by calling attention in these reports to the evidence available, it provides the final reason for such apparently non-medical measures as free meals.

### THE BADGE QUESTION.

Much interest has been aroused by the article published last week on "War Service Badges for Hospital Staffs," and we continue to receive inquiries and correspondence with reference to the matter. It should therefore be pointed out that the question of providing such badges is not only one which is receiving attention from hospital managers, but that certain members of Red Cross bodies, ambulance workers, and the like, are also possessed of a desire to be supplied with them. The motive in many cases is not confined to a wish to be protected from the importunity of recruiting agents. There is also the desire for an ostensible sign that the wearer is engaged on "war work," and is proud to be able to signalise the fact in this way. The great point to remember is that if badges are not to be overdone they should be granted, if at all, only by responsible authorities, and confine themselves to a bare statement of the organisation with which the wearer is connected.

### INCREASE OF MEDICAL STAFF AT A POOR-LAW INFIRMARY.

We have frequently drawn attention to the inadequate medical staffs provided for our large Poor-Law infirmaries. The following, therefore, is of interest. The St. Pancras Board of Guardians have considered a report of their infirmary committee, which recommends that an additional assistant medical officer (male or female) be appointed for the south infirmary and house, as in the committee's opinion the present staff is too small. It is satisfactory to note that, despite some opposition by members of the Board, the infirmary committee's recommendation was adopted, and the Local Government Board will be asked to sanction this additional appointment. The Poor-Law Institutions Order created much extra work for medical officers of Poor-Law institutions, and now that so many general hospitals are being utilised for our wounded sailors and soldiers, the acute cases demanding treatment at Poor-Law infirmaries tend to increase. These cases, naturally, require more constant attention than many of the "chronics."

### ANOTHER COMMISSION FOR A HOSPITAL OFFICER.

MR. F. HARTLEY, secretary's assistant at the Manchester Children's Hospital, has been gazetted as lieutenant, Army Service Battalion, 9th Lincolnshire Regiment.

### THE LATE CAPTAIN GARNIER AND EAST SUSSEX HOSPITAL.

AT the recent annual meeting of the East Sussex Hospital the Mayor of Hastings (Councillor E. A. Hocking) referred to the death of Captain J. W. Garnier, the former secretary, who has died of his wounds. The Mayor proposed that they should place on record their sorrow at his loss, and those present rose in their places. We need not repeat here his record of service which we gave in THE HOSPITAL of June 5, but we may take this opportunity to remind Mr. Hocking and the committee of the East Sussex Hospital that a worthy custom has now been established by which those hospitals whose secretaries fall in action put up a tablet to record the fact. When we first made this suggestion in reference to the death at the Front of Mr. A. E. Thomas, formerly secretary of the Hampstead General Hospital, it was quickly acted upon, and a tablet to his memory was lately unveiled by the Grand Duke Michael of Russia, the president of the hospital. We publish a full account of the proceedings in THE HOSPITAL of May 8, together with an illustration of the memorial tablet. While Mr. Thomas was the first hospital secretary to fall in the great war, it is only right and fitting that other hospitals should follow the example of the Hampstead General Hospital, and record by a tablet of this kind every instance in which a hospital secretary gives his life at the Front.

### THE BRITISH HOSPITAL IN MOSCOW.

AN interesting account of the work now being done among the Russian wounded in connection with this institution appeared in the *Daily Chronicle* last Tuesday. The writer, who signs himself "F. D. W.," states that the hospital consists of two wards on different floors, each of which accommodates about seventy-five wounded. He draws attention to the work which is being done by the ladies of the British colony, in the hands of which the whole organisation is centred. The writer describes the appearance of the wards, and the "rude wooden beds," and gives an impression of the nurses and the medical officer at work. He remarks that "no praise can be too high for the unselfish manner in which the ladies of the British colony are doing their share in the great struggle." Such accounts of the work which our fellow-subjects are performing in Russia cannot but stimulate our interest and help us to realise the vast stage on which the present conflict is being fought out.

### A REDUCTION IN A DRUG ACCOUNT.

IT is somewhat unusual to find an institution which has been able to reduce its expenditure on drugs, but the Surrey Dispensary, of Great Dover Street, Southwark, can lay claim to having done so. The dispensary was founded in 1777 for gratuitously attending women and for providing medical and surgical aid to the poor inhabitants of the Borough of Southwark. The annual report states that a very great increase has taken place in the price of many drugs and medicines, but with careful management it has been found possible to reduce the dispensary drug account so that the cost of medicine per patient during 1914 was 3⅝d. per head, as against 5d. per head in 1913. It might be helpful to know by what means this interesting decrease has been effected. The extent of the reduction may be realised from the fact that 12,491 letters, including thirty-six midwifery letters, were issued to patients and renewed during the twelve months, whilst the resident medical officer made 976 visits to patients.

### THIS WEEK'S DRUG MARKET.

THE drug market continues active, and in some departments there is increased activity. The general tendency of prices is still in an upward direction, and in only a few cases are there any indications of impending reductions in value. It seems not unlikely that glycerine may be advanced in price; indeed, it is rather surprising that quotations have remained unchanged for so long considering that such colossal quantities are required for the manufacture of explosives. This country is in a very satisfactory position as regards the supplies of glycerine; in Germany, on the other hand, supplies are so restricted that chemists are only allowed to sell it in very small quantities, the Government having taken possession of the whole of the output. Salicylates seem likely to advance still further in price; in the value of synthetic drugs generally there is practically no change, but prices are firmly maintained. Cod-liver oil is unchanged in price, and the demand is slow at the moment. Fair business continues to be done in quinine at firm rates. Quicksilver is again dearer, and calomel and corrosive sublimate and other salts of mercury have consequently advanced in price again. Methylated spirit is dearer. In Italian produce there is little change, but cream of tartar and citric acid seem likely to advance still further in price. The opium market is the centre of some interest; in the event of an early forcing of the Dardanelles the market would no doubt be affected very considerably, but failing that it is difficult to see how any reduction in the present high value of opium can be expected. The demand for morphine and codeine and other opiates is becoming larger than ever owing to the large requirements of the Army Medical Service. Balsam of Peru is getting still scarcer and dearer; it is being used as a remedy against lice.

